# A novel mutation causing mild, atypical fumarylacetoacetase deficiency (Tyrosinemia type I): a case report

**DOI:** 10.1186/1750-1172-4-28

**Published:** 2009-12-15

**Authors:** David Cassiman, Renate Zeevaert, Elisabeth Holme, Eli-Anne Kvittingen, Jaak Jaeken

**Affiliations:** 1 Center for Metabolic Diseases, Leuven University Hospitals, Leuven, Belgium; 2 Department of Clinical Chemistry, Salhgrenska Hospital, Göteborg, Sweden; 3 Institute of Clinical Biochemistry, University of Oslo, 0027 Oslo, Norway

## Abstract

A male patient, born to unrelated Belgian parents, presented at 4 months with epistaxis, haematemesis and haematochezia. On physical examination he presented petechiae and haematomas, and a slightly enlarged liver. Serum transaminases were elevated to 5-10 times upper limit of normal, alkaline phosphatases were 1685 U/L (<720), total bilirubin was 2.53 mg/dl (<1.0), ammonaemia 69 μM (<32), prothrombin time less than 10%, thromboplastin time >180 s (<60) and alpha-fetoprotein 29723 μg/L (<186). Plasma tyrosine (651 μM) and methionine (1032 μM) were strongly increased. In urine, tyrosine metabolites and 4-oxo-6-hydroxyheptanoic acid were increased, but succinylacetone and succinylacetoacetate - pathognomonic for tyrosinemia type I - were repeatedly undetectable. Delta-aminolevulinic acid was normal, which is consistent with the absence of succinylacetone. Abdominal ultrasound and brain CT were normal.

Fumarylacetoacetase (FAH) protein and activity in cultured fibroblasts and liver tissue were decreased but not absent. 4-hydroxyphenylpyruvate dioxygenase activity in liver was normal, which is atypical for tyrosinemia type I. A novel mutation was found in the FAH gene: c.103G>A (Ala35Thr). *In vitro *expression studies showed this mutation results in a strongly decreased FAH protein expression.

Dietary treatment with phenylalanine and tyrosine restriction was initiated at 4 months, leading to complete clinical and biochemical normalisation. The patient, currently aged 12 years, shows a normal physical and psychomotor development.

This is the first report of mild tyrosinemia type I disease caused by an Ala35Thr mutation in the FAH gene, presenting atypically without increase of the diagnostically important toxic metabolites succinylacetone and succinylacetoacetate.

## Introduction

Type I tyrosinemia (OMIM +276700), also called hepatorenal tryosinosis, is a severe inborn metabolic disease affecting the tyrosine degradation pathway. It often presents with liver disease or liver failure with predominant bleeding tendency, Fanconi syndrome and/or rickets (for a comprehensive review, see [1]). Type I tyrosinemia is caused by a mutation in the gene encoding for the fumarylacetoacetate hydrolase or fumarylacetoacetase (FAH) enzyme, an enzyme in the tyrosine degradation pathway. Deficiency of this enzyme causes intracellular accumulation of fumarylacetoacetate (FAA), a tyrosine-derived metabolite upstream of the deficient FAH enzyme. FAA is thought to be genotoxic and therefore carcinogenic. Intracellular FAA is rapidly degraded to succinylacetone (SA) and succinylacetoacetate (SAA), which are also thought to be carcinogenic. Patients with type I tyrosinemia can also develop acute neuropathic pains or polyneuropathy with respiratory failure, reminiscent of acute porphyria, due to inhibition of heme-synthesis at the level of aminolevulinic acid dehydratase, by the produced toxic metabolites of tyrosine degradation [1].

The diagnosis of type I tyrosinemia is based on the presence of liver disease, kidney disease and/or rickets, increased tyrosine and methionine in plasma and the presence of SA in urine and blood and SAA in urine. In addition to SA and SAA, the presence of 4-oxo-6-hydroxyheptanoic acid in urine has also been described as pathognomonic [2]. The presence of SA and SAA is considered pathognomonic for the disease. Up till now, no type I tyrosinemias without SA or SAA in urine have been described [1]. The diagnosis of type I tyrosinemia is confirmed by measurement of FAH enzyme activity in cultured fibroblasts (or on liver tissue) and/or detection of disease-causing mutations in the FAH gene. In total, 44 FAH mutations are listed in the Human Genome Mutation database http://www.hgmd.cf.ac.uk.

Type I tyrosinemia is treated with a protein-restricted diet, amino acid supplements low in tyrosine, phenylalanine and methionine, and nitisinone. Nitisinone is a drug that inhibits 4-hydroxyphenylpyruvate dioxygenase, an enzyme upstream of FAH, thereby preventing the formation of the toxic compounds FAA, SA and SAA [1]. Typically, the activity of this enzyme is already reduced in type I tyrosinemia, presumably be feedback-inhibition of the accumulating toxic end-products in the diseased patient.

The natural history of the typical disease is an evolution to liver failure, cirrhosis with hepatocellular carcinoma, end-stage renal failure, acute neuropathic pains and hypertrophic cardiomyopathy.

The evolution of the disease has improved considerably since the introduction of nitisinone treatment, but - depending on the age at diagnosis and start of treatment - development of liver and kidney disease is not entirely excluded. Especially the occurrence of hepatocellular carcinoma is a dreaded complication.

## Materials and methods

### Isolation of RNA and Northern blotting

The isolation of total RNA from fibroblasts, electrophoresis, blotting and hybridisation with a 32P-labelled single stranded FAH probe, was performed as described previously [3]. The membranes were reprobed with pig β-actin cDNA as control.

### **Western blotting**

Was performed according to Berger et al. [4].

### Genomic PCR, sequencing and restriction analysis

A genomic DNA product of 252 bp across FAH exon 2 was PCR amplified with primers 5'-GGACTCTTCAATAGACAGG-3' (sense, intron 1) and 5'-CCACAGTAAGTGCCACTGAG-3' (antisense, intron 2) and used for direct sequencing (Thermo Sequenase radiolabeled terminator cycle sequencing kit from Amersham, The Netherlands).

For enzyme restriction analysis a 175 bp PCR product across the mutation was amplified by 30 cycles of 94°C for 30 sec and 60°C for 60 sec, followed by 3 min final extension at 72°C. The primers were 5'-ATCTTCCTCCTAGCCAAGACCGAGGATAGGGGTG-3' (sense) and 5'-CCACAGTAAGTGCCACTGAG-3' (antisense). A mismatch (underlined) in the sense primer together with the Ala35Thr mutation creates a restriction site for Bst EII. The PCR assay contained, in a total volume of 100 μl, 5-10 μl of cell lysate, 10 μl of PCR buffer, 0.2 mM of each dNTP, 40 pmol of each primer, 2 μmol MgCl2 and 1 μl Dynazyme DNA polymerase. The PCR products were digested with either HaeIII or Bst EII and electrophoresed on 3.5% NuSieve minigels. With HaeIII the 175 bp PCR product without mutation is digested into fragments of 142 and 33 bp. With Bst EII the product with the Ala35Thr mutation is digested into fragments of 141 and 34 bp.

### Mutagenesis and expression analysis

Mutagenesis using the Altered Sites In Vitro Mutagenesis System (Promega), expression analysis using TnT Coupled Reticulocyte Lysate System (Promega), determination of FAH activity of the translated products, and autoradiography of ^35^S-methionine-labeled translation products subjected to PAGE were performed as previously described [3].

### **Liver enzyme activities and urinary and blood organic acid analysis**

Were performed as described previously [2,5].

## Case Description

A 4 month old boy, born at 37 weeks of gestation, to non-consanguineous Caucasian parents presented at the emergency department with epistaxis, hematemesis and hematochezia. He was born following a pregnancy complicated with maternal oedema and preterm contractions. On physical exam at 4 months, petechiae, a subconjunctival bleeding in the left eye and a friable haemangioma on the inside of the left cheek were noted. The liver was slightly enlarged. Serum transaminases were elevated to 5-10 times upper limit of normal, alkaline phosphatase was 1685 U/L (<720), total bilirubin was 2.53 mg/dl (<1.0), ammonaemia was 69 μM (<32), prothrombin time was less than 10% (>70), thromboplastin time was >180 s (<60) and alpha-fetoprotein was 29723 μg/L (<186).

Hepatitis caused by drugs, hepatitis viruses or hepatotropic viruses was excluded.

Plasma tyrosine (651 μM) and methionine (1032 μM) were strongly increased. In urine, tyrosine metabolites and 4-oxo-6-hydroxyheptanoic acid were increased, but SA and SAA were repeatedly undetectable. Delta-aminolevulinic acid was normal, which is consistent with the absence of SA. There was no tubulopathy, skeletal X-ray showed no rickets. Abdominal ultrasound and brain CT were normal.

Treatment with restriction of natural protein and amino acid formula low in phenylalanine and tyrosine was initiated at 4 months. Nitisinone was initially not started because of absence of the supposedly toxic metabolites of tyrosinemia type I and complete clinical and biochemical normalisation on diet (normal liver tests, alpha-fetoprotein, coagulation tests).

**Figure 1:**
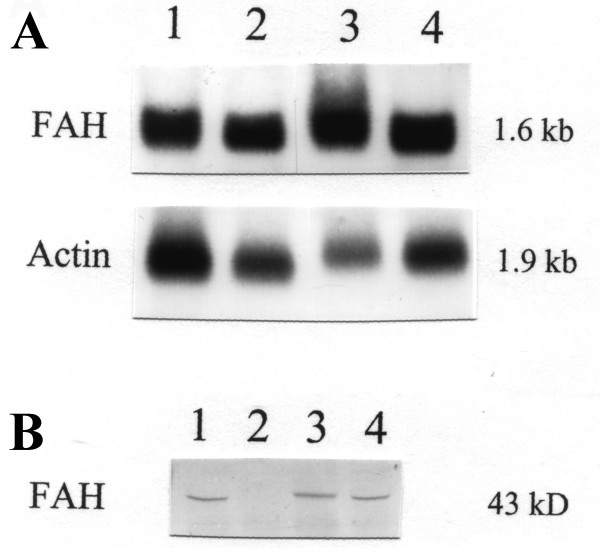
**Expression of fumarylacetoacetase (FAH) mRNA and protein in fibroblast extracts**. A: Northern blot (1). Patient mRNA in lane 2 and 4, control sample in lane 1 and 3. Upper panel: probed with a ^32^P-labeled FAH cDNA. Lower panel: reprobed with pig beta-actin. B: Western blot (2). Patient protein in lane 2, control samples in lanes 1, 3 and 4. FAH mRNA is expressed in the patient's fibroblasts, but FAH protein could not be detected.

**Figure 2:**
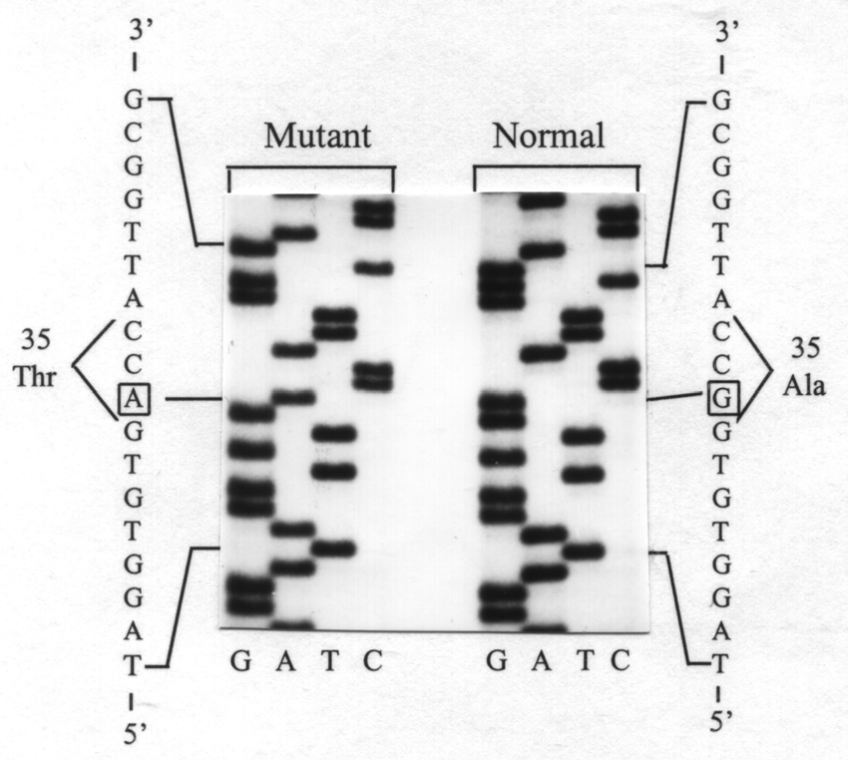
**Identification of the Ala35Thr mutation**. PCR-amplified genomic DNA was sequenced and revealed a G to A transition in the first nucleotide of codon 35 in exon 2 of the FAH gene, leading to a substitution of Ala by Thr.

**Figure 3:**
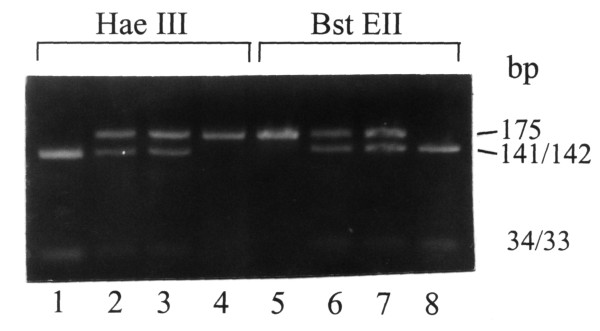
**The Ala35Thr mutation can be confirmed by restriction analysis**. Lanes 1 and 5: control. Lanes 2 and 6: the patient's mother. Lanes 3 and 7: the patient's father. Lanes 4 and 8: the patient. Restriction analysis with HAEIII and BstEII, on DNA extracted from blood, confirms heterozygosity for the Ala35Thr mutation in both parents and homozygosity in the patient.

**Figure 4:**
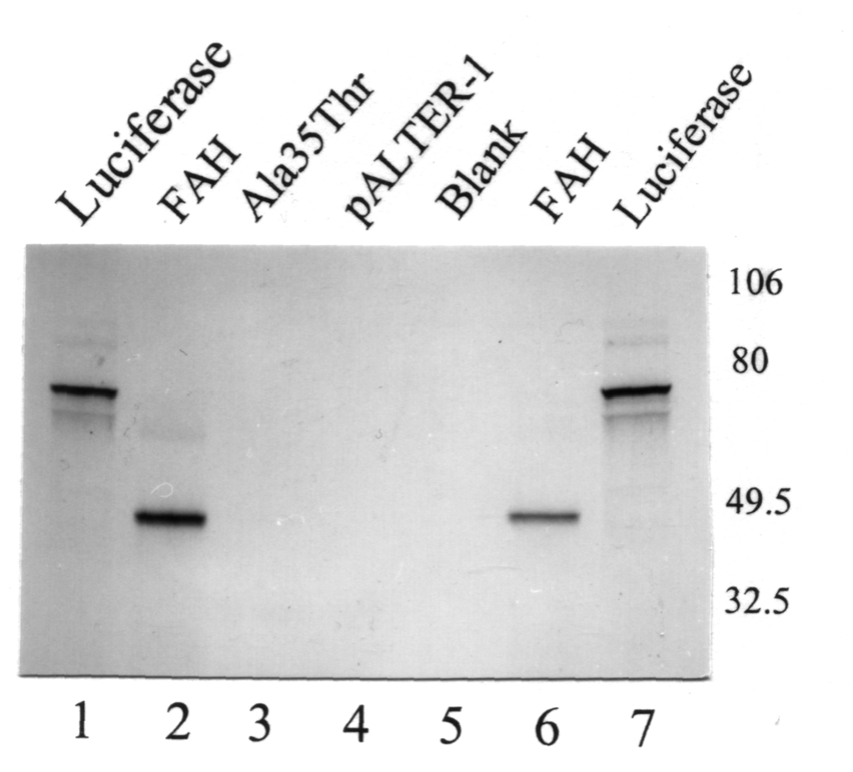
**Expression analysis of Ala35Thr mutated FAH**. Using the TnT Coupled Reticulocyte Lysate System (Promega) (2), wild-type FAH and mutated FAH were expressed. An autoradiography of ^35^S-methionine-labeled translation products separated by polyacrylamide gel electrophoresis is shown. Lanes 1 and 7: luciferase expression (control). Lanes 2 and 6: expression of 43 kDa FAH control. Lane 3: expression of FAH mutant with Ala35Thr. Lane 4: plasmid without insert. Lane 5: no DNA control. Ala35Thr mutation of the FAH gene leads to absence of protein expression.

Fumarylacetoacetase (FAH) mRNA in fibroblasts was normal (Fig. 1). FAH protein and activity in cultured fibroblasts (Fig. 1) and liver tissue (Table 1) were decreased but not absent. 4-hydroxyphenylpyruvate dioxygenase activity in liver was normal, which is atypical for tyrosinemia type I. A novel mutation was found in the FAH gene: c.103G>A (Ala35Thr). (Fig. 2) Homozygosity for this mutation in the patient, heterozygosity in both his parents, was shown by restriction analysis with HAEIII and BstEII. (Fig. 3) *In vitro *expression studies showed this mutation resulted in absence of FAH protein expression. (Fig. 4)

**Table 1:** Liver enzyme activities

	Biopsy I	Biopsy II	Tyr type III	Controls
FAH	2.2	1.8	33	41 (38.8-46.4)
4-HPD	11	8.7	0.2	15 (10.8-18.4)
ASAT	3472	2669	2490	1731,1641
ALAT	700	810	1340	665, 1034
LDH	3315	2223	3420	3755, 3045

Column 1 shows the result of a first biopsy in the presented patient (at diagnosis), the second column of a confirmatory biopsy in the same patient. Column 3 shows the results in a type III tyrosinemia, for comparison. The last column shows control values. FAH is clearly decreased, but measurable in both biopsies in our patient (approx. 5% of normal). 4-HPD is unexpectedly not equally decreased as would be expected in type I tyrosinemia, also contrasting with the value measured in the type III tyrosinemia biopsy. The fact that 4-HPD is not decreased, is compatible with the absence of SA and SAA in our patient. ASAT, ALAT and LDH are internal controls, demonstrating the quality of the tissue samples used.

FAH: fumarylacetoacetate hydrolase; 4-HPD: 4-hydroxyphenylpyruvate dioxygenase; ASAT: aspartate transaminase; ALAT: alanine transaminase; LDH: lactate dehydrogenase

Because of clinical and biochemical normalisation (table 2 and 3), the diet was terminated at the age of 22 months. Parameters remained normal during follow-up. At the age of 6 years the diet was reintroduced together with nitisinone treatment (0.5 mg/kg, later increased to 1.0 mg/kg) after the identification of a hypointense lesion on MRI of the liver. The lesion was considered suspicious and was resected. Pathological examination showed a region of disturbed microcirculation, no arguments for malignancy. Electron microscopy of the tissue surrounding the resected lesion showed enlarged mitochondria with paracrystalline inclusions together with hypertrophy of the Golgi apparatus. These findings are non-specific, but suggestive of toxicity. Annual follow-up liver MRI up till now (12 years of age) has shown no focal lesions. The patient's psychomotor development is normal. His renal function is normal. He is still on diet and nitisinone therapy.

**Table 2:** Urinary organic acid analysis results

Age	4 months	5 months	8 months	18 months
Succinylacetone	0.2	0.1	0.1	≈ 0.1
Succinylacetoacetate	≈ 0.1	<0.1	≈ 0.3	<0.1
4-oxo-6-hydroxyheptanoic acid	4	1	2	8
creatinine, mmol/L	3.1	1.6	3.1	3.2

A significantly increased excretion of aberrant tyrosine metabolites was detected, with 4-oxo-6-hydroxyheptanoic acid as the dominant metabolite. The excretion of phenolic tyrosine metabolites was not significantly increased: in the first sample 4-hydroxyphenylpyruvate was 7 mmol/mol creatinine, which can be considered as marginally increased (ref value in newborns <10, in infants <5). In later samples, we found 4-hydroxyphenylpyruvate <1 mmol/mol creatinine (reference value <5) and 4-hydroxyphenyllactate 2 mmol/mol creatinine (reference value < 10). These values are in accordance with a normal 4-hydroxyphenylpyruvate dioxygenase activity (Table 1).

**Table 3:** Blood and urine biochemistry and enzymatic activities

	Age		4 months	5 months	8 months	18 months	30 months	6 years Start NTBC	6 years and 1 month
	Unit	Ref range							
**BLOOD**									
PBG synthase in RBC	nkat/g Hb	0.58-1.25	**--**	0.51	0.17	0.05	0.06	0.11	0.870
Succinylacetone in plasma	μmol/L	< 0.1	0.88	0.13	<0.1	0.33	0.38	0.27	<0.10
α-Fetoprotein in serum	μg/L	<12	**--**	28000	50	11	<5	<5	<5
Tyrosine in plasma	μmol/L	50-130	693	36	73	96	112	130	381
Phenylalanine in plasma	μmol/L	40-120	196	57	72	70	83	89	45
Methionine in plasma	μmol/L	20-50	1300	400	30	20	39	34	15
**URINE**									
5-Aminolevulinic acid	mmol/mol creatinine	0-3	6.9	5.3	8.8	25	17	7.9	3.5
Succinylacetone	mmol/mol creatinine	<1	<1	<1	<1	<1	1.1	0.3	<0.1
Succinylacetoacetate	mmol/mol creatinine	<1	<1	<1	<1	<1	<1	<0.3	<0.1
4-oxo-6-hydroxyheptanoic acid	mmol/mol creatinine	<1	4	1	2	8	4.2	6	<0.1
NTBC in serum	μmol/L		--	--	--	--	--	--	28.7

## Discussion

We report here a case of a mild Type I Tyrosinemia, presenting typically with liver disease (jaundice, bleeding tendency), increased tyrosine and methionine in plasma, but absence of the pathognomonic markers SA and SAA from urine. The diagnosis of type I tyrosinemia is supported by the demonstration of 4-oxo-6-hydroxyheptanoic acid in urine, a strongly decreased but measurable FAH enzyme activity in liver tissue and fibroblasts and the demonstration of a novel mutation in the FAH gene (Ala35Thr). In the first 6 years following diagnosis, the condition was successfully managed with a tyrosine/methionine-restricted diet, resulting in rapid and complete resolution of liver disease.

Although apparently mild in presentation, we do not consider it likely that the disease only became clinically apparent, by the presence of an intercurrent problem, e.g. a viral or xenobiotic-induced hepatitis, since the most likely causes of hepatitis were excluded in this patient, by standard hepatitis work-up. The cause of the hepatitis episode in this patient, with a striking coagulopathy and highly increased α-fetoprotein typical of Type I Tyrosinemia, was therefore most likely the suggested mild type I tyrosinemia.

In type I tyrosinemia patients with a cirrhotic liver, liver nodules demonstrating a reversion to the normal FAH genotype and showing normal FAH enzyme activity have been described [5]. The reversion is thought to result from a combination of mutation pressure exerted by the toxic compounds produced in these patients (FAA, SA, SAA), combined with a high rate of liver cell proliferation. In the patient reported here, there was no advanced liver disease or generalised nodular transformation at the time of biopsy, so the measured residual FAH enzyme activity is considered a genuine residual activity (of about 5%). In the transgenic lethal albino mice, 0.3%-4.3% of normal expression of FAH was shown to be compatible with a normal life-span of the mice [6], which is concordant with the described evolution of our patient so far.

In our opinion, the normal SA and SAA in this patient is due to the residual activity of the FAH enzyme. The absence of the typical suppression of both the aminolevulinic acid dehydratase and 4-hydroxyphenylpyruvate dioxygenase, supports the observation that SA and SAA are low. Inversely, absence of suppression of these enzymes in the presence of type I tyrosinemia, supports the supposition that it is indeed these compounds that normally suppress their activity in typical type I tyrosinemia.

In view of the complete resolution of liver disease in this patient under dietary restrictions, and absence of the metabolites considered toxic in this disease, no nitisinone treatment was started. The evolution over the first 6 years of life was entirely uneventful. Nitisinone was started from 6 years to date (current age 12 years), because of a cancer scare (identification and resection of a suspicious liver lesion that proved not malignant). In addition, the lack of hard data on the absence of evolution to end-stage liver disease with hepatocellular carcinoma, or to tubulopathy in this hitherto undescribed type I tyrosinemia inspired prudency in the treating physicians.

Finally, since there apparently can be tyrosinemia presentations without SA in urine and without increased aminolevulinic acid, this has to be taken into account in the interpretation of those newborn screening programs, testing for SA [7] or aminolevulinic acid [8] in urine. The patient presented here would not have been detected by such screening program and nevertheless has proven type I tyrosinemia.

In conclusion, we present a patient homozygous for a previously undescribed mutation in the FAH gene (Ala35Thr), leading to a mild type I tyrosinemia and probably amenable with diet. This mild tyrosinemia presented without urinary SA and SAA.

## List of abbreviations

FAA: fumarylacetoacetate; FAH: fumarylacetoacetate hydrolase or fumarylacetoacetase; SA: succinylacetone; SAA: succinylacetoacetate.

## Consent

Written informed consent was obtained from the patient's parents for publication of this case report and accompanying images. A copy of the written consent is available for review by the Editor-in-Chief of this journal.

## Competing interests

The authors declare that they have no competing interests.

## Authors' contributions

DC, RZ and JJ were involved in the clinical follow-up of the patient, the data analysis and interpretation and drafted the manuscript. EH and EAK performed the biochemical studies, the genetic studies and the interpretation of the results. All authors read and approved the final manuscript.
